# Prevalence, awareness, and associated risk factors of hypertension in older adults in Africa: a systematic review and meta-analysis protocol

**DOI:** 10.1186/s13643-017-0585-5

**Published:** 2017-10-04

**Authors:** William K. Bosu, Justice M. K. Aheto, Eugenio Zucchelli, Siobhan Reilly

**Affiliations:** 1 West African Health Organisation (WAHO), 175 Avenue Ouezzin Coulibaly, 01 BP 153, Bobo-Dioulasso, Burkina Faso; 2 School of Public Health, University of Ghana, LG 13, Legon, Accra Ghana; 3 Division of Health Research, Faculty of Health & Medicine, Lancaster University, Furness Building, Lancaster, LA1 4YG UK

**Keywords:** Older adults, Hypertension, Risk factors, Determinants, Awareness, Africa

## Abstract

**Background:**

The health of older persons has not been a major priority in many African countries. Hypertension is one of the common health problems of older persons. However, there is little information on the prevalence of hypertension in older adults in Africa. This is in spite of the fact that Africa has the highest age-standardized prevalence of hypertension in the world. We therefore present this protocol to conduct a systematic review and meta-analysis on the prevalence of hypertension and the level of its awareness among older persons living in Africa.

**Methods:**

Major databases (EMBASE, MEDLINE, Academic Search Complete, CINAHL, PsycINFO) and unpublished literature will be searched to identify population-based studies on hypertension in adults aged 50 years and older living in Africa. Eligible articles are those which use the 140/90-mmHg cutoff to diagnose hypertension and were published from 1980 to present. We will exclude subjects in restricted environments such as patients and refugees. Articles will be independently evaluated by two reviewers to determine if they meet the inclusion criteria. They will also evaluate the quality of included studies using a validated tool by Hoy and colleagues for prevalence studies. The main outcome is the prevalence of hypertension while the explanatory variables include demographic, socio-economic, dietary, lifestyle and behavioural factors. Effect sizes in bivariate and multivariate analyses will be presented as odds or prevalence ratios. We will explore for heterogeneity of the standard errors across the studies, and if appropriate, we will perform a meta-analysis using a random-effects model to present a summary estimate of the prevalence of hypertension in this population.

**Discussion:**

The estimates of the prevalence, the risk factors and the level of awareness of hypertension could help in galvanizing efforts at prioritizing the cardiovascular health of older persons in Africa.

**Systematic review registration:**

PROSPERO CRD42017056474

**Electronic supplementary material:**

The online version of this article (10.1186/s13643-017-0585-5) contains supplementary material, which is available to authorized users.

## Background

Hypertension is a major risk factor for cardiovascular diseases, accounting for 52.5% of all strokes in Africa compared with 38.8% in the industrialized region [1]. The World Health Organization (WHO) estimates that its African Region had the highest age-standardized prevalence of adult hypertension in the world in 2008 (38.1% in men and 35.5% in women) [2]. The mean blood pressure levels in sub-Saharan Africa (SSA) increased significantly between 1975 and 2015 to levels that are among the highest in the world [3]. Before-and-after studies as well as estimates from systematic reviews also show significant increases in the prevalence of hypertension in the same rural or urban populations over time in Africa [4–6]. Important features of hypertension in Africa include young age of onset of complications [7], high prevalence of target organ damage [8–11], poor knowledge about the disease in the general population and among hypertensive persons [12, 13], use of alternative therapy [14, 15], low levels of detection and control [16–18] and clustering of risk factors [19] and co-morbidities [18].

Older persons have higher levels of mean blood pressure and hypertension [20]. For example, a recent systematic review estimated that the pooled prevalence of hypertension in adults aged 65 + years (61.0%) in SSA was 2.5 times that among persons aged 18–64 years (24.4%) [20]. An epidemiological model showed that the pooled prevalence of hypertension in Africa in the elderly ranges from two to four times than that in 40 years or younger [21]. Older persons aged 50 + years are more likely than younger adults in urban and rural Africa to have hypertension or to report risk factors for chronic non-communicable diseases (NCDs) [22, 23].

Older persons in Africa suffer multi-morbidity, with a majority having hypertension in addition to other health problems such as angina, arthritis, asthma, cataract, chronic obstructive pulmonary disease, depression, diabetes, edentulism, cognitive impairment, obesity and stroke [24–27]. NCDs, particularly cardiovascular diseases, are the commonest cause of medical admissions among the elderly in Africa [28]. The treatment and control rates of hypertension in older adults in Africa are generally lower than those in high-income countries [18, 29–31].

The population of Africa is ageing with the proportion aged 60 years and above (60 +) projected to increase from 5.2% in 2000 to 8.9% in 2050 and 19.6% in 2100 [32]. SSA will experience the fastest growth rate from the 1950s to the 2040s in the world [33]. The changing demographics, accompanied by an epidemiologic transition favour an increase in chronic NCDs. The Institute of Health Metrics and Evaluation estimates that the proportion of total deaths due to NCDs in Africa that occurred among persons aged 50 + years increased from 67.3% in 1990 to 71.1% in 2015 [34]. The share of all NCD deaths among those aged 50 + years also increased from 66.5 to 71.6% over the same period.

Among persons aged 50–69 years in Africa, the leading risk factors for disease burden, measured in disability-adjusted life years (DALYs) lost, are high systolic blood pressure (SBP), high body mass index and high fasting plasma glucose. The proportion of deaths attributable to cardiovascular diseases in Africa remained stable at 29.1% in 1990 and 28.9% in 2015. The proportion of DALYs attributable to cardiovascular diseases decreased slightly over the same period was from 23.1 to 22.1%. These statistics vary between countries. In Ghana, for example, cardiovascular diseases were the leading health problems in persons aged 50–69 years in 2015 accounting for 30.3 and 24.5% of total deaths and DALYs lost respectively.

It is not often appreciated that hypertension is preventable even in old age through lifestyle changes [35]. For example, promoting light or moderate physical activity has been shown to reduce mortality and heart attacks in older men [36]. Treatment of hypertension in older adults prevents cardiovascular complications and improves their quality of life [37]. The beneficial impact of lowering blood pressure on cardiovascular disease and mortality is not limited to younger or middle-aged adults [38].

There are several reasons why a systematic review on the prevalence of hypertension in older adults in Africa is needed. While a number of systematic reviews of hypertension have been undertaken at country level [39–42], sub-regional [19] and continental levels [17, 43, 44] and recently among children and adolescents [45], there is little information among the older adult population in Africa. To date, only one systematic review with meta-analysis of the prevalence of hypertension in older adults in Africa has been published [46]. Unlike our proposed review which defines older age as 50 years and older in line with other studies [23, 31, 47], the Kaze et al. study used a threshold of 55 years [46] and reported a pooled prevalence of hypertension of 55.2% among persons aged 55 years and older. There was substantial heterogeneity (*I* ^2^ = 95.5%) across the studies analysed, due probably in part to their method of study selection. Our review will analyse studies primarily targeting older persons, whereas their review extracted data pertaining to older persons as a subset from any study of the general population. These differences in study selection could lead to substantial differences in the number and sample sizes in the final set of studies that are analysed. For example, two papers by Addo et al. [48, 49] conducted respectively in rural subjects aged 18–99 years and urban civil servants aged 25–68 years would not be eligible in our study whereas they contributed three data points from the age groups 55–64 years, ≥ 55 years and ≥ 65 years in the Kaze et al. [46] study.

Besides the differences in the set of studies to be analysed, we expect the different study selection methods to yield different study sample sizes, both of which affect heterogeneity across studies. In household surveys in Africa, the older age groups tend to be under-represented. Consistent with this observation, the 55 + years age group constituted only about 21% of the total sample size in the Kaze et al. [46] review. As an example of the differences in the sample size to be evaluated, whereas our review on the prevalence of hypertension will include all (100%) the 3840 South Africans aged 50 years and older in an eligible study [50], the Kaze et al. [46] review included only the age group 60 years and older which represented 55.9% of the total sample. Further, we expect that the inclusion of studies published prior to the year 2000 will yield additional relevant studies.

Restricting our studies to those that were conducted specifically in older persons will provide a better basis to make policy proposals relating to this age group than the Kaze et al. review in which the included studies covered a mix of age groups. A study conducted with the aim to assess the difference in the prevalence of hypertension between formal and informal urban areas in an African city [51] or one that aimed to estimate the prevalence of hypertension and associated risk factors in civil servants [49] would hardly be expected to yield findings that are directly relevant to older persons. Thus, we expect the profile of subjects in the included studies between our review and the published review [46] to be significantly different owing to the differences in the age groups, time period and inclusion criteria.

### Review question

The review questions are as follows:

1. What is the prevalence and trends in hypertension in adults aged 50 years and older living in Africa?
2. What are the factors associated with hypertension in older adults in Africa?
3. What is the level of awareness of high blood pressure among older adults in Africa?

## Methods

### Protocol and registration

This protocol complies with the Preferred Reporting Items for Systematic Reviews and Meta-analyses (PRISMA-P) guidelines (Additional file 1) [52]. It is registered on PROSPSERO international prospective register of systematic reviews (http://www.crd.york.ac.uk/PROSPERO), registration number CRD42017056474 [53]. In the event of any amendment to this protocol post-publication, then the date and the rationale for the amendments will be published in the PROSPERO register in a way that enables these changes to be tracked.

### Inclusion criteria

The following criteria should be met by studies to be included in the analysis:

1. Types of studies: population-based cross-sectional studies and follow-up studies published from January 1980 to present which report prevalence or incidence of hypertension in older adults.
2. Types of articles: both published and unpublished articles will be eligible for inclusion. Conference abstracts which provide adequate information on the sample size, data collection methods and analysis as well as the prevalence of hypertension will be considered.
3. Population: apparently healthy adults aged 50 years and older living in Africa
4. Outcome measures: the main outcome measure is the prevalence or incidence of hypertension. Secondary outcomes include the grades of hypertension, the proportion of hypertensives detected and the presence of co-morbidities.
5. Setting/context: only studies of persons living in Africa will be included. Multi-country or multi-region studies will be included if there is distinct analysis of data pertaining to African countries.

### Exclusion criteria

We will exclude the following studies:

1. Type of population:
  - Studies involving older adults in a restricted population such as those who are unwell, attending hospitals or are in institutions for chronic or mental illness
  - Studies involving older persons in refugee settings
  - African migrants living outside the African region
2. Types of articles: Review articles, case reports and expert opinion commentaries
3. Type of outcome measure: studies whose main outcome is self-reported hypertension or non-systemic hypertension such as pulmonary hypertension
4. Geographical area: studies conducted outside the African region

### Literature search

We will search the major electronic databases (EMBASE and MEDLINE via Ovid and Academic Search Complete, CINAHL and PsycINFO via EBSCOhost) and repositories (African Index Medicus and African Journals Online). Using the PICO’s approach [54, 55], we will search the literature based on four concepts: the population (older adults), the type of measure (prevalence or incidence), outcome (hypertension or raised blood pressure) and the geographic setting (Africa). In EMBASE and MEDLINE databases, the search terms relating to three concepts will include major medical subject headings (MeSH) and free-text words (Table 1). The Boolean operators ‘OR’ will be used with synonyms within each concept and then the search results for the different concepts combined with the ‘AND’ operator. The aim is to maximise the yield of the relevant papers. Some of the search terms will be used in truncated formats to permit wider and different sets of the term to be applied simultaneously. Regarding the geographic context (Africa), the different regions and the individual countries of the continent will be entered separated by the ‘OR’ operator.

**Table 1:** Search method in EMBASE and Ovid MEDLINE

#	Searches
1	exp hypertension/ep, et, pc [Epidemiology, Etiology, Prevention]
2	(Hypertensi* or "blood pressure" or cardiovascular or cardiometabolic).ab.
3	1 or 2
4	exp prevalence/
5	exp incidence/
6	(Prevalence or proportion or survey or descriptive or cross-sectional or cohort or longitudinal or "attributable fraction" or incidence).sh.
7	4 or 5 or 6
8	exp Africa/
9	indian ocean/ or exp mauritius/ or exp reunion/ or exp seychelles/
10	(africa* or algeria* or angola* or benin* or botswana* or burkina* or burundi* or cameroon* or canary islands or cabo verde or cape verde* or central african republic or chad or comoros or comores or congo* or democratic republic of the congo or djibouti or egypt* or equatorial guinea* or eritrea* or ethiopia* or gabon* or gambia* or ghana* or guinea* or bissau or cote d'ivoire or kenya* or lesotho or liberia* or libya* or jamahiriya or jamahiryia or madagascar or malawi* or mali* or mauritania* or mauritius or morocc* or mozambi* or mocambique or namibia* or niger* or nigeria* or reunion or rwanda* or sao tome or senegal* or seychelles or sierra leone* or somalia* or south africa* or st helena or sudan* or swazi* or tanzania* or tanganyika or togo* or tunisia* or uganda* or western sahara or zaire* or zambia* or zimbabwe*).sh.
11	8 or 9 or 10
12	3 and 7 and 11
13	limit 12 to ("middle age (45 to 64 years)" or "all aged (65 and over)") [Limit not valid in Embase; records were retained]
14	limit 13 to humans
15	limit 14 to yr="1980 -Current"

Regarding the fourth concept, the study population, we will apply the limits available in the database to select the subset of studies pertaining to those aged 50 years and older. Besides the proceedings and abstracts from conference that will be obtained from the above databases, we will search Google Scholar and ProQuest for other grey literature presented as dissertations or unpublished reports. Where possible, we will contact the authors for the full results of conference abstracts. The bibliographies of the selected papers that the selected databases yield will also be hand-searched to locate further articles of interest.

### Selection of studies

**Fig. 1:**
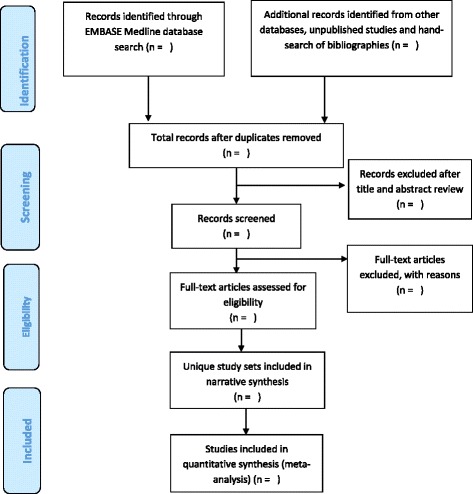
Flow chart for selection of papers. Charts the selection of articles in the review in line with the Preferred Reporting Items for Systematic Review and Meta-analysis (PRISMA) Framework

Duplicate studies from the different electronic databases, which emerge after using the above search strategy, will be removed through the EndNote programme. The titles and abstracts of the articles of results that emerge from the search strategy will be screened independently by two reviewers to exclude obviously non-relevant papers (Fig. 1). Then the full-text versions of the remaining potentially eligible articles will be retrieved and further assessed independently by two reviewers to determine if they satisfy the inclusion criteria. Any discrepancies will be resolved through consensus. Corresponding authors will be contacted to provide copies of papers whose full text is not available online. In line with the PRISMA framework, the reasons for exclusion at full-text screen level will be documented (Fig. 1) [52].

The total number of unique studies from all sources which fulfill the inclusion criteria will be recorded. The list of studies that were excluded from analysis and the reasons for their exclusion will be reported as supplementary information.

### Data extraction

Data will be extracted using a standardised extraction form in Microsoft Excel (Additional file 2). The data to be captured will include the publication details, language of the paper, study period, study location, study objectives, geographic setting, study design, study period, characteristics of participants, sample size and sampling technique, explanatory and outcome variables, data analysis and the major findings. Specific data such as the demographic and socio-economic characteristics, any anthropometric measurements, blood pressure measurement procedures, the overall prevalence and grades of hypertension, and the effect sizes of statistically significant variables will be extracted. The variables that are associated with hypertension will be categorised into psychosocial, economic, social, dietary, behavioural and lifestyle factors [56]. Where multiple papers pertaining to the same study population and site are encountered, we will extract data from the most informative paper(s) but they will be linked together as one unique study paper. The earliest year of publication will be adopted if multiple papers of the same study are published at different times.

In line with the Seventh Report of the Joint National Committee on the Prevention, Detection, Evaluation and Treatment of High Blood Pressure (JNC VII), hypertension will be defined as having a SBP ≥ 140 mmHg and/or diastolic pressure (DBP) ≥ 90 mmHg or using antihypertensive drug therapy in the previous 2 weeks [57]. Those who report being told of a previous diagnosis of hypertension by a health professional would be considered as being aware of their hypertension. The three grades of hypertension will be defined as grade 1 (SBP 140–159 mmHg or DBP 90–99 mmHg), grade 2 (SBP 160–179 mmHg or DBP 100–109 mmHg) and grade 3 (SBP ≥ 180 mmHg or DBP ≥ 110 mmHg) [58]. The number of risk factors in the same individuals and the presence of co-morbidities such as diabetes and chronic kidney disease will be documented.

### Assessment of the quality of studies

The quality of included studies will be assessed using a risk of bias tool specifically designed and validated for cross-sectional studies [59]. The tool assesses both internal and external validity based on responses to ten questions. As with the study selection, two different investigators will independently evaluate the quality of the included papers and characterize them as having a high, moderate or low risk of bias. Any discrepancies will be resolved by mutual consent and discussion.

### Data analysis and synthesis

The characteristics of the study participants will be summarised in tables using descriptive statistics. The main outcomes are hypertension, the grades of hypertension, and the proportion of controlled hypertension. The strength of the association between these outcomes and the various demographic, socio-economic and lifestyle factors in bivariate and multivariable analysis will be reported. The confounders adjusted in each analysis will be documented for each study.

The prevalence data and risk factors from the different studies will be summarised using narrative synthesis. The prevalence will be summarised as a proportion, and summary measures of risk factor-hypertension association, presented as odds ratios. If statistically appropriate, prevalence estimates from studies with common definitions of hypertension will be pooled together to provide a single summary estimate through a random-effects meta-analysis [60].

We will report the crude and pooled estimates of the prevalence of hypertension in 10-year time periods over the period 1980–2017 in order to provide trends [19, 41]. In addition, we will analyse the temporal trends in the prevalence of hypertension using cumulative meta-analysis [46, 61].

We will explore heterogeneity through the inspection of forest plots as well as with the chi-squared test on Cochran’s Q (alpha set at 0.1) statistic [62] and an *I* ^2^ test. For the latter, the cut-points for low, moderate and high degrees of heterogeneity will be set at 25, 50 and 75% respectively [63]. Separate sub-group analyses will be performed to assess the causes of any heterogeneity and, if statistically appropriate, to estimate the pooled prevalence of hypertension by sex, age group, geographic locality, study design and year of publication (by decades). We will perform a sensitivity analysis to examine the effect of excluding studies with high-risk of bias on the overall prevalence and relationships. The presence of reporting bias will be determined through funnel plot asymmetry if more than 10 eligible studies are included and Egger’s test [64]. Data analysis will be performed using the Stata version 14 and RevMan 5.3 programmes.

The overall quality of evidence will be based on an adaptation of the Grading of Recommendations Assessment, Development and Evaluation (GRADE) guidelines [65]. The GRADE criteria for the evaluation of evidence are risk of bias, consistency (heterogeneity), directness (generalizability), precision (statistical significance of effect measures) and publication bias. The quality of evidence is graded as high, moderate, low or very low. For observational studies, the quality of evidence starts off as low [66]. The risk of bias will be derived from the Hoy et al. tool designed for prevalence studies [59].

## Discussion

To our knowledge, this is the first published protocol to systematically estimate the prevalence of hypertension and its associated factors in the adult population aged 50 years and older in Africa. Furthermore, our review has several other strengths. The search strategy is comprehensive in including published and unpublished literature. The four-decade period covered by the review is sufficiently long to establish patterns and relationships with the outcome. The reviewers recognize that bias can occur even in apparently well-conducted studies [67]. Hence, the review will evaluate the risk of bias in the primary studies. In order to reduce bias, two researchers will independently screen and agree on the study papers to be included and assess their quality [52, 68]. To ensure maximum yield, no language restrictions will be applied.

As with other reviews [21, 44, 69], we do not specifically include ‘awareness’ and ‘risk factors’ in the search terms. The heterogeneity in the study population, blood pressure measurement protocols and study methods may limit the meta-analysis or generalizability. Narrative synthesis would be used if meta-analysis is not appropriate. Another limitation is that most of the studies will be cross-sectional and so will not be best suited to assess temporal sequence between a risk factor and the outcome.

The findings will be submitted for publication in peer-reviewed open access journals as well for presentation at a scientific conference. They will be shared with the health departments of the African Union and the Regional Economic Communities of Africa to promote discussion among regional and national policy makers. The paper will add to the knowledge of the burden of hypertension and its associated lifestyle and behavioural practices in this group of older adults who are largely neglected in many African countries. The findings will assist countries to commit to favourable policies and programmes for healthy ageing, in line with the World Health Organization’s global strategy on ageing and health [70].

This protocol presents a comprehensive and reproducible approach to systematically estimate the prevalence of hypertension in the older adult population of Africa. It tries to conform with the best practices at each stage of the protocol development in order to assure high-quality output. It is expected that the findings of the systematic review would galvanize national and regional efforts to improve the cardiovascular health of this often-neglected population in Africa.

## Additional files

Additional file 1: PRISMA-P 2015 checklist 01 08 2017. Description of data: Contains the line numbers for the different items on the PRISMA-P checklist. (DOCX 33 kb)

Additional file 2: Data extraction form HTN protocol. Data extraction form for studies on the prevalence of hypertension in older adults in Africa. Contains the data extraction form. (DOCX 38 kb)

## Abbreviations

BP: Blood pressure
DALY: Disability-adjusted life years
DBP: Diastolic blood pressure
JNC: Joint National Committee
NCDs: Non-communicable diseases
PRISMA: Preferred Reporting Items for Systematic Review and Meta-Analysis
PROSPERO: International prospective register of systematic reviews
SBP: Systolic blood pressure
SSA: Sub-Saharan Africa
WHO: World Health Organization
