# Supplementary material for: Prevalence, awareness, and associated risk factors of hypertension in older adults in Africa: a systematic review and meta-analysis protocol
Source: Syst Rev. 2017 Oct 4;6:192. doi: 10.1186/s13643-017-0585-5 (PMC5628476; doi:10.1186/s13643-017-0585-5)
Supplement: Supplementary file 2 — Data extraction form HTN protocol. Data extraction form for studies on the prevalence of hypertension in older adults in Africa. Contains the data extraction form. (DOCX 38 kb) [file 13643_2017_585_MOESM2_ESM.docx]

*Additional File 2: Data extraction form for studies on the prevalence of hypertension in older adults in Africa*

| **No.** | **Category** | **Description** | **Possible Values** | | | **Needed to assess paper for inclusion?** |
| --- | --- | --- | --- | --- | --- | --- |
| 1 | Unique Paper ID |  |  | | |  |
| 2 | Extraction | Date of extraction |  | | | Yes |
|  |  | Name of extractor |  | | |  |
| 3 | Reference | Name of Lead author |  | | | Yes |
|  |  | Corresponding author's email |  | | |  |
|  |  | Title |  | | |  |
|  |  | Journal |  | | |  |
|  |  | Year of Publication |  | | |  |
|  |  | Type of Publication | original article, abstract, comment, conference paper, dissertation | | |  |
| 4 | Extra articles | Potentially eligible studies identified in reference list | Yes, No, Not sure | | |  |
|  |  | Which numbered references could be retrieved for review? |  | | |  |
| 5 | Language | Language in which article is published | English, French, Other | | | Yes |
| 6 | Study period | Date data collection started |  | | |  |
|  |  | Date data collection ended |  | | |  |
|  |  | Duration of data collection (enrolment to last follow-up) |  | | |  |
| 7 | Setting | Is paper part of a multi-country publication? | Yes, No | | | Yes |
|  |  | Country | name of country, multi-country | | |  |
|  |  | Sub-region in Africa | northern, southern, western, eastern, central | | |  |
|  |  | Province |  | | |  |
|  |  | City |  | | |  |
|  |  | Geographic Coverage | National, sub-national (province, district, town, suburb) | | |  |
|  |  | Type of location | Institutional, community, other | | |  |
|  |  | Type of residence | Urban, peri-urban, rural | | |  |
| 8 | Study objectives | Main objective/aim/purpose of study |  | | |  |
|  |  | Is hypertension main objective of the study? | Yes, No, Not sure | | |  |
| 9 | Study design | Type of study | Cross-sectional, cohort, intervention | | |  |
| 10 | Sampling | Type of participants | specify age-group covered | | | Yes |
|  |  | Is sampling technique clearly described in sufficient detail? | Yes, No, Not sure | | |  |
|  |  | Sampling technique | Convenience, simple random, stratified random, cluster, multistage | | |  |
|  |  | Sample representative? | Yes, No, Not sure | | |  |
|  |  | Indicate if sample size calculated | Calculated, Arbitrary, Not reported | | |  |
|  |  | Sample size | Overall total, number of men and women | | |  |
|  |  | Determine if sample size adequate from own perspective |  | | |  |
| 11 | Main Outcome of interest | If prevalence of hypertension in eligible age group is reported | Yes, No | | | Yes |
| 12 | **Decision on inclusion criteria** | Initial decision on inclusion for further review | **Include** | **Exclude** | **Not sure** | **Do not proceed if article is excluded** |
|  |  | If excluded, reason for exclusion |  | | |  |
|  |  | For those papers for which initial decision to include was uncertain, state final decision after discussion | **Include** | **Exclude** | **Unresolved** |  |

| **No.** | **Category** | **Description** | **Possible Values** | | |
| --- | --- | --- | --- | --- | --- |
| 13 | Ethics | Ethical approval obtained? | Yes, no, not reported | | |
|  |  | If yes, who provided ethical clearance? |  | | |
|  |  | Ethics involved proxy consent? | Yes, no, not reported | | |
|  |  | If yes, on whose behalf? |  | | |
| 14 | Follow-up design | If longitudinal, what was length of follow-up? | Cross-sectional, cohort, quasi-experimental | | |
|  |  | How follow-up was done | home visits, clinic visits; frequency of visits | | |
|  |  | % loss to follow-up |  | | |
| 15 | Data collection technique | How data collected |  | | |
|  |  | Anthropometry | What measurements? | | |
|  |  | Other (e.g. laboratory) measurements |  | | |
| 16 | Participation rate | % of eligible sample who participated in study |  | | |
|  |  | Reasons for withdrawals and exclusions |  | | |
| 17 | Anthropometry | % of participants with anthropometric data |  | | |
|  |  | Who did the measurements? | Health professional, trained persons, not reported | | |
|  |  | How overweight/obesity defined? |  | | |
|  |  | How was weight measured? |  | | |
|  |  | How was height measured? |  | | |
|  |  | How were waist and hip circumferences measured? |  | | |
|  |  | Where measurements taken? |  | | |
|  |  | If measurements conform to standard procedures? | Yes, No, Not reported, unclear | | |
| 18 | BP measurement protocol | No. of participants enrolled with BP measured |  | | |
|  |  | Personnel taking BP |  | | |
|  |  | No. of visits | Single, multiple, not stated | | |
|  |  | If multiple, which subjects re-visit? | all subjects; those with elevated BP; known hypertensives | | |
|  |  | Interval between visits | No. of days or weeks between visits | | |
|  |  | Frequency of readings per visit |  | | |
|  |  | Initial rest time (mins) |  | | |
|  |  | Interval between multiple readings (mins) |  | | |
|  |  | Reading used in analysis |  | | |
|  |  | Cuff size |  | | |
|  |  | Posture of subject |  | | |
|  |  | Part of body on which BP taken |  | | |
|  |  | Device |  | | |
|  |  | Where BP measurements taken? | subjects' home; health centre; public facility e.g. school; mixed, other | | |
|  |  | Quality control measures instituted, if any |  | | |
|  |  | % of enrolled subjects whose BP measurement |  | | |
| 19 | Measurement of outcomes | How hypertension defined? | Cut-off BP; those on anti-hypertension medication | | |
|  |  | If subjects on medication as hypertensives, regardless of BP reading | Yes, No, Not reported, unclear | | |
|  |  | How long should subjects have been on medication? | Yes, No, Not reported, unclear | | |
|  |  | How uncontrolled BP defined |  | | |
| 20 | Type of analysis | If multivariable analysis done? |  | | |
|  |  | Type of multivariable analysis | Multiple regression, logistic regression, multilevel regression, other | | |
|  |  | Variables adjusted for in the model |  | | |
|  |  | How blood pressure treated in analysis? | Continuous, binary, multilevel | | |
| 21 | Study population characteristics |  | **Male** | **Female** | **Total** |
|  |  | Frequency (number) |  |  |  |
|  |  | Age range |  |  |  |
|  |  | Mean age ± s.d |  |  |  |
|  |  | Median age |  |  |  |
|  |  | No. aged 70+ years |  |  |  |
|  |  | Number currently married |  |  |  |
|  |  | No. with no education |  |  |  |
|  |  | No. not in active employment |  |  |  |
|  |  | No. with health insurance |  |  |  |
|  |  |  | **Urban** | **Rural** | **Total** |
|  |  | Frequency (number) |  |  |  |
|  |  | Age range |  |  |  |
|  |  | Mean age ± s.d |  |  |  |
|  |  | Median age |  |  |  |
| 22 | Risk factors |  | **Male** | **Female** | **Total** |
|  |  | No. of current smokers |  |  |  |
|  |  | No. taking alcohol |  |  |  |
|  |  | Heavy drinkers |  |  |  |
|  |  | physically inactive or with low physical activity |  |  |  |
|  |  | insufficient fruit & vegetable intake |  |  |  |
|  |  |  |  |  |  |
|  |  |  |  |  |  |
| 23 | Findings - anthropometric measures |  | **Male** | **Female** | **Total** |
|  |  | Frequency |  |  |  |
|  |  | Mean weight (kg) |  |  |  |
|  |  | Mean height (cm) |  |  |  |
|  |  | Mean BMI |  |  |  |
|  |  | Mean waist circumference |  |  |  |
|  |  | Mean hip circumference |  |  |  |
|  |  | Mean waist-to-hip ratio |  |  |  |
| 24 | Weight categories - BMI | % underweight |  |  |  |
|  |  | % normal weight |  |  |  |
|  |  | % overweight 25.0 - 29.9 kg/m^2^ |  |  |  |
|  |  | % obese ≥30.0 kg/m^2^ |  |  |  |
|  |  | % severely obese ≥40.0 kg/m^2^ |  |  |  |
|  |  | % central obesity (WHR >0.9 in men, >0.85 in women) |  |  |  |
|  |  | % increased waist circumference WC ≥88cm in women; ≥102cm in men |  |  |  |
| 25 | Findings - blood pressure | number with valid BP readings |  |  |  |
|  |  | mean systolic BP (SBP) ± SD |  |  |  |
|  |  | mean diastolic BP (DBP) ± SD |  |  |  |
| 26 | Findings - hypertension | Number whose hypertension status assessed |  |  |  |
|  |  | Number with raised BP (hypertension) |  |  |  |
|  |  | % normal pressure |  |  |  |
|  |  | % pre-hypertension (high normal) |  |  |  |
|  |  | % grade 1 (SBP 140–159 mm Hg or DBP 90–99 mm Hg) |  |  |  |
|  |  | % grade 2 (SBP 160–179 mm Hg or DBP 100–109 mm Hg) |  |  |  |
|  |  | % grade 3 (SBP ≥180 mm Hg or DBP ≥110 mm Hg) |  |  |  |
|  |  | isolated systolic hypertension |  |  |  |
|  |  | isolated diastolic hypertension |  |  |  |
|  |  | % any hypertension (Grades 1 - 3) |  |  |  |
|  |  | % hypertension including those on antihypertensive medication |  |  |  |
| 27 | Hypertension by residence |  | **Urban** | **Rural** | **Total** |
|  |  | Number with raised BP (hypertension) |  |  |  |
|  |  | % any hypertension (Grades 1 - 3) |  |  |  |
|  |  | % hypertension including those on antihypertensive medication |  |  |  |
| 28 | Age-specific mean SBP | (specify age-groups) | **Male** | **Female** | **Total** |
|  |  | Age group 1 |  |  |  |
|  |  | Age group 2 |  |  |  |
|  |  | Age group 3 |  |  |  |
|  |  |  |  |  |  |
| 29 | Age-specific mean DBP | Age group 1 |  |  |  |
|  |  | Age group 2 |  |  |  |
|  |  | Age group 3 |  |  |  |
|  |  |  |  |  |  |
| 30 | Age-specific Hypertension | Age group 1 |  |  |  |
|  |  | Age group 2 |  |  |  |
|  |  | Age group 3 |  |  |  |
|  |  |  |  |  |  |
| 31 | Awareness of hypertension | Number previously aware that they have hypertension |  |  |  |
|  |  | % awareness |  |  |  |
| 32 | Detection | number of newly identified participants with raised BP/hypertension |  |  |  |
|  |  | % of all hypertensives who were newly diagnosed or identified |  |  |  |
| 33 | Treatment | number of persons with hypertension on treatment |  |  |  |
|  |  | % of all hypertensives currently on medication, regardless of whether new or old hypertensives |  |  |  |
|  |  | % of known hypertensives currently on medication |  |  |  |
| 34 | Control of BP | number of persons with hypertension on treatment whose BP is controlled |  |  |  |
|  |  | number of persons with hypertension, whether or not on treatment, whose BP is controlled |  |  |  |
|  |  | % number of persons with hypertension on treatment whose BP is controlled |  |  |  |
| 35 | Co-morbidities | List co-morbidities and specify prevalence | **Male** | **Female** | **Total** |
|  |  | Diabetes |  |  |  |
|  |  | Chronic kidney disease |  |  |  |
|  |  | Target organ damage |  |  |  |
|  |  | Metabolic syndrome |  |  |  |
|  |  | Disability |  |  |  |
|  |  | Depression |  |  |  |
|  |  |  |  |  |  |
|  |  |  |  |  |  |
| 36 | Explanatory variables (univariate analysis) | Specify which variables had statistically significant relationships with hypertension in the crude analysis, effect size, interval estimates |  | | |
|  |  | Specify which variables had non-statistically significant relationships with hypertension in the crude analysis, effect size, interval estimates |  | | |
| 37 | Effect sizes of statistically significant associations in crude analysis | Specify variables, the comparator, and their effect sizes along with confidence intervals |  |  |  |
|  |  |  |  |  |  |
|  |  |  |  |  |  |
|  |  |  |  |  |  |
|  |  |  |  |  |  |
|  |  |  |  |  |  |
|  |  |  |  |  |  |
|  |  |  |  |  |  |
|  |  |  |  |  |  |
|  |  |  |  |  |  |
|  |  |  |  |  |  |
|  |  |  |  |  |  |
|  |  |  |  |  |  |
|  |  |  |  |  |  |
|  |  |  |  |  |  |
| 38 | Clustering of risk factors | Prevalence of 2 risk factors |  |  |  |
|  |  | % ≥2 risk factors (daily tobacco use, frequent heavy drinker, low physical activity, low fruit and vegetable intake, central obesity, general obesity, hypertension) |  |  |  |
|  |  | Prevalence of 3 risk factors |  |  |  |
|  |  | Prevalence of 4+ risk factors |  |  |  |
| 39 | Determinants (independently associated with outcome in multivariate analysis) | Specify the effect size measure used | Specify type of measure (prevalence ratio, odds ratio, correlation coefficient | | |
|  |  | Type of multivariable analysis | Multiple regression, logistic regression, multilevel regression, other | | |
|  |  | Are the comparison (reference) categories same as in the crude analysis (Q. 37)? | Yes, No | | |
|  |  | If no, describe changes in the variables made for the multivariate analysis |  | | |
|  |  | Specify total number of different multivariate models, if multiple |  | | |
|  |  | Variables adjusted for in the model |  | | |
|  |  | Positive determinants | List variables positively associated with outcome, with effect sizes and confidence intervals | | |
|  |  | Negative determinants | List variables significantly and negatively associated with outcome, with effect sizes and confidence intervals | | |
|  |  | Non-significant variables | List variables not significantly associated with outcomes | | |
| 40 | Main conclusions | What are the key conclusions of the paper? |  | | |
| 41 | Study limitations and strengths | What limitations are reported by the authors? |  | | |
|  |  | What strengths are reported by the authors? |  | | |
| 42 | Paper reviewer comments | Are the paper reviewers' comments published? | Yes, no | | |
| 43 |  | *Comments of reviewer A* |  | | |
|  |  | *Comments of reviewer B* |  | | |
|  |  | *Comments of reviewer C* |  | | |
| 44 | General notes | Write any additional notes that you have |  | | |
